# Event and Comment

**Published:** 1916-09

**Authors:** 


### DENTAL REGISTER

Vol. LXX SEPTEMBER, 1916 No. 9

### EVENT AND COMMENT

PROFESSIONAL INSTRUCTION IN DENTISTRY. The editor of *Oral Hygiene* in the August issue indulges in some comments on the showing made by some schools in respect to the standing of their graduates before the various State Boards of Examiners, as is shown by the Tabulation Committee of the National Association of Dental Examiners. This report undoubtedly gives a fairly good estimate of the value of the instruction given by the several schools, at the same time it may be that there are extenuating circumstances which would give these results a different interpretation if known. He seems to think that if a dozen schools can show only from three to ten per cent. of failures there is no good reason why the other three dozen should not do as well, and he believes there is no good reason for the existence of a school which for a number of years has had from twenty-five to fifty per cent. of its graduates fail to pass the State Board examinations. His remedy is that teachers in dental colleges must be educated men in all departments and must make teaching their principal business, rather than an incident to the practice of their professions. From Brother Belcher's viewpoint this argument is not only strong but convincing. But if he knew as much about conducting a dental college as he does about editing he would see that there are conditions that confront the schools complained of that he does not understand. In the first place there is not a dental college in the country that could pay sufficient salaries to equip itself with a faculty of expert professional teachers who would devote all their time to teaching. Teachers are notoriously so inadequately paid that young men are not willing to qualify for such positions when they can with much less effort and more freedom build a practice in a short time that will be much more remunerative and providential.

Then there are so many hazards to be overcome, or at least that are likely to interrupt one's happiness that most men prefer to work out their careers independent of the capriciousness of those in control of an institution. For this reason we shall not soon have any considerable number of devoted professional teachers. Some of the schools whose graduates fail most constantly have men on their faculties that are eminent for learning and skillfulness in their professions; but they are to a considerable extent dependent on their own efforts in a professional way for the financial support they need to maintain themselves socially. We do not mean to infer that we have no adequate teaching in our dental colleges, neither do we want to leave the inference that there is no material difference in the quality of instruction given in the several schools; most of our schools are well managed and have competent instructors, but for financial reasons, largely, our schools are not as well equipped for teaching as they should be. For this reason obviously it can not be expected that such schools can maintain an ideal curriculum. The only hope we can see for the future is to secure sufficient endowment for our dental schools as shall provide ample means for paying salaries that shall justify the material sacrifices required of men who elect a teaching profession. If our schools were thus endowed and they were associated with large universities so that their permanence would be assured, they could be more economically administered, and incidentally men would be attracted by the honors of association with other educators. There are real joys to be had from giving one's life to teaching and most real teachers are satisfied to give their best efforts to a work which at best holds few attractions in the way of monetary rewards.

At the present time there is a well-defined tendency to better our dental educational facilities, and there has been a marked improvement during the past ten years. The field of dentistry is constantly broadening and much more is exacted of a teaching faculty now than formerly; and, it may be that the profession is in advance of the instruction in our college, one might so infer from the fact that so many college graduates fail to come up to the standards of our examiners who are all practitioners and not teachers, but our teachers and colleges are most important factors in all professional advancement.

The Tabulation Committee is doing good work as it is revealing our weakness. We feel assured that such an exposition will result in more careful and competent work in our schools, and that when we come to see our duty clearly we shall make such changes as will remedy the weakness of our system of education, but it may require patience and time for its accomplishment. We are just now facing some important problems in dental education. We need more well qualified dentists, and we are making it more difficult for men to enter the profession. We shall need to make the work of the profession more inviting if we are to attract properly educated men and women. Shall we encourage the establishment of dental schools in connection with established universities of high educational standards, or endeavor to make of our privately owned dental colleges teaching institutions of equivalent rank? The first proposition seems to be the natural and most promising method, and in our judgment will command the respect of those seeking a professional education, and will win the confidence and support of the profession more definitely and permanently than the second, we believe.

